# Sexual selection impacts brain anatomy in frogs and toads

**DOI:** 10.1002/ece3.2459

**Published:** 2016-09-12

**Authors:** Yu Zeng, Shang Ling Lou, Wen Bo Liao, Robert Jehle, Alexander Kotrschal

**Affiliations:** ^1^ Key Laboratory of Southwest China Wildlife Resources Conservation (Ministry of Education) China West Normal University Nanchong Sichuan China; ^2^ School of Environment & Life Sciences University of Salford Salford UK; ^3^ Zoological Institute Stockholm University Stockholm Sweden

**Keywords:** anuran, brain anatomy, brain size evolution, comparative analysis, courtship types, mating system, PGLS, testes mass

## Abstract

Natural selection is a major force in the evolution of vertebrate brain size, but the role of sexual selection in brain size evolution remains enigmatic. At least two opposing schools of thought predict a relationship between sexual selection and brain size. Sexual selection should facilitate the evolution of larger brains because better cognitive abilities may aid the competition for mates. However, it may also restrict brain size evolution due to energetic trade‐offs between brain tissue and sexually selected traits. Here, we examined the patterns of selection on brain size and brain anatomy in male anurans (frogs and toads), a group where the strength of sexual selection differs markedly among species, using a phylogenetically controlled generalized least‐squared (PGLS) regression analyses. The analysis revealed that in 43 Chinese anuran species, neither mating system, nor type of courtship, or testes mass was significantly associated with relative brain size. While none of those factors related to the relative size of olfactory nerves, optic tecta, telencephalon, and cerebellum, the olfactory bulbs were relatively larger in monogamous species and those using calls during courtship. Our findings support the mosaic model of brain evolution and suggest that while the investigated aspects of sexual selection do not seem to play a prominent role in the evolution of brain size of anurans, they do impact their brain anatomy.

## 1 Introduction

Most theories of vertebrate brain size evolution consider natural selection as the main evolutionary force shaping its diversification (Striedter, 2005). Indeed, a great number of comparative and experimental studies demonstrated the interplay between natural selection and brain size evolution (Aiello & Wheeler, 1995; Gonzalez‐Voyer & Kolm, 2010; Kotrschal et al., 2013; Liao, Lou, Zeng, & Merilä, 2015; Sol, Székely, Liker, & Lefebvre, 2007; Tsuboi et al., 2015). Additionally, within the last years, evidence across a wide range of taxa has accumulated that sexual selection might also affect brain size evolution (Boogert, Fawcett, & Lefebvre, 2011; Fitzpatrick et al., 2012; Garamszegi, Eens, Erritzøe, & Møller, 2005; García‐Peña, 2013; Gonzalez‐Voyer & Kolm, 2010; Kotrschal et al., 2015; Lemaitre, Ramm, Barton, & Stockley, 2009; Pitnick, Jones, & Wilkinson, 2006). The subset of studies that provide empirical evidence that sexual selection and brain size are associated, base their argument on the rationale that better cognitive skills afforded by larger brains increase the chances of obtaining mates (Boogert et al., 2011; Garamszegi et al., 2005). We adhere to the broad definition of “cognition” as comprising “all mechanisms that invertebrates and vertebrates have for taking in information through the senses, retaining it, and using it to adjust behavior to local conditions” (Kotrschal & Taborsky, 2010; Shettleworth, 2010).

In contrast to the studies suggesting positive effects of sexual selection on brain size, other studies propose that sexual selection should restrict brain size evolution on the grounds of a trade‐off. The development of costly sexual traits may limit the energy available for the development of the brain (Fitzpatrick et al., 2012; Gonzalez‐Voyer & Kolm, 2010; Pitnick et al., 2006). However, several studies did not detect any relationships between the investigated aspects of sexual selection and brain size, such as testes size (Lemaitre et al., 2009; Schillaci, 2006), or sexual coloration (Kotrschal et al., 2013). The degree to which sexual selection impacts brain evolution is therefore still an open question.

Within the field of sexual selection, the mating system of a species has been suggested to drive the evolution of its brain size (García‐Peña, 2013; Pitnick et al., 2006; Schillaci, 2006). Again, two opposing hypotheses predict the evolutionary relationship between the mating system and the brain size of vertebrates. The “sexual conflict hypothesis” argues that the ongoing struggle between males and females to subvert the reproductive investment of the other sex is cognitively demanding (Arnqvist & Rowe, 2005). As a result, promiscuous species will have relatively larger brains than species with genetic monogamy (Rice & Holland, 1997). Conversely, the “expensive sexual tissue” hypothesis contends that more intense sexual selection will constrain the evolution of brain size again due to energetic trade‐offs with costly sexual organs, ornaments, or armaments (Garamszegi et al., 2005; Pitnick et al., 2006). Empirical evidence only partially supports this hypothesis. For instance, while Pitnick et al. (2006) found that bat species with larger brains have smaller testes than bats with smaller brains, Dechmann and Safi (2009) did not find such a relationship for another set of bat species. Besides whole brain size, the mating system can also affect the size of some brain regions. In primates, for example, the intensity of male–male competition is negatively associated with neocortex size and the neocortex is larger in monogamous species (Schillaci, 2008). Similarly, in cichlid fishes, the region analogous to the primates neocortex, the telencephalon, is larger in monogamous compared to polygamous species (Pollen et al., 2007). Like the neocortex in porimates, the fish telencephalon integrates more complex cognitive processes; both are likely selected for by the cognitive challenges of long‐term pair bonds, which are typical for monogamous species. In contrast, a later study on a greater number of cichlid species did not find any association between sexual selection and telencephalon size (Gonzalez‐Voyer & Kolm, 2010).

Courtship is often crucial in sexual selection (Andersson, 1994) and courtship calls and mate searching are two common behaviors during courtship. They give cues of the male's reproductive status during female mate choice as well as during competition among males (Duellman & Trueb, 1986). Yet despite recognition that species differences in courtship behavior are modulated via differences in distinct cell groups in different parts of the brain and that these cell groups have independent effects (Balaban, 1997), the relationship between the type of courtship and the evolution of the brain and its regions remains enigmatic.

Here, we examined the associations between relative brain size, the size of five main brain regions, and three fundamental traits of sexual selection among 43 anuran species. Within this group, it is already established how phylogeny and ecology contribute to variation in brain morphology (Liao et al., 2015). Here, we investigated the effect of the mating system (monandry vs. polyandry), the courtship type (attracting mates with courtship calls vs. searching for mates quietly), and the intensity of sexual selection (using testes mass as proxy) on brain morphology by means of phylogenetically controlled generalized least‐squared (PGLS) regression analyses. Anurans are an excellent model system to test these relationships because of their diverse breeding systems, ecology, and life histories (Byrne & Roberts, 2012; Duellman & Trueb, 1986). The extreme variance in the degree of sexual selection across species (Byrne, Simmons, & Roberts, 2003) allowed us to comprehensively test whether mating system and courtship type are associated with differences in brain size and the size of brain regions (viz. olfactory nerves, olfactory bulbs, telencephalon, optic tectum, and cerebellum). Olfactory nerves were included because these are also used by most anurans to process olfactory information, often called smaller/accessory olfactory bulbs, and they may represent a distinct olfactory system (Taylor, Nol, & Boire, 1995).

There is debate whether vertebrate brain regions evolve in a mosaic or concerted manner, that is, whether brain regions increase and/or decrease with overall brain size or whether specific selection pressures can select for size changes of brain regions independently (see e.g., Barton & Harvey, 2000; Finlay, Darlington, & Nicastro, 2001; Gonzalez‐Voyer, Winberg, & Kolm, 2009; Liao et al., 2015; Yopak et al., 2010). Our data set allows testing for those alternatives. If anuran brain regions evolve in a mosaic manner in response to sexual selection, we would expect single regions to vary independently. Concerted evolution would be indicated if overall brains but not single regions would vary in size. For the relationship between brain size and the chosen traits of sexual selection, the hypotheses above give clear, yet at times opposing, predictions. However, for brain region volumes, it is difficult to make such predictions. This is so because the function of the separate brain regions is still only partly understood and because single regions sometimes have multiple functions (Striedter, 2005). However, the olfactory bulbs and optic tectum mainly integrate olfactory and visual information, respectively; those regions are generally more prominent in species with better olfactory and visual acuity (Butler & Hodos, 2005). Both vision and olfaction play prominent roles in anuran mate choice (Liao & Lu, 2009, 2010), we therefore predict that in species searching for mates (instead of calling), those regions should be larger to facilitate mate search efficiency. For volumes of the other regions, we avoid making predictions and treat this part of the analysis as a prospect to identify the regions of the brain that are most affected by sexual selection.

## 2 Materials and Methods

### 2.1 Field sampling

We collected a total of 200 adult male individuals from 43 anuran species during the breeding seasons 2007–2013 from the Hengduan Mountains of China. Individuals were transferred to the laboratory and then killed by double‐pithing (Mi et al., 2012, Jin et al., 2016). We obtained volumetric measures of overall brain size and the five major different brain regions (viz. olfactory nerves, olfactory bulbs, telencephalon, optic tectum, and cerebellum) for all individuals (Table 1). Medulla volume was not determined because pithing damaged the structural integrity of the brain stem; whole brain mass is not affected by this method, however (Jiang et al., 2015). All specimens were preserved in 4% phosphate‐buffered formalin for tissue fixation. After 2 weeks to 2 months of preservation, body size (snout‐vent length: SVL) was measured to the nearest 0.01 mm with calipers. Brains and testes were dissected out and weighed to the nearest 0.1 mg with an electronic balance. The number of days samples spent in the buffered formalin did not affect relative brain weight (Liao et al., 2015) and testes mass (Zeng, Lou, Liao, & Jehle, 2014). We chose the species on the basis of diversity of courtship behavior and mating system, access to samples, as well as on the basis of available phylogenetic information.

**Table 1:** Species, number of samplings, average snout‐vent length (SVL: mm), body mass (g), testes mass (mg), brain size (mm^3^), and volume of different brain parts (mm^3^)

Species	N	SVL	Body mass	Brain size	Olfactory nerves	Bulbus olfactorium	Telencephalon	Optic tectum	Cerebellum	Testes mass	Mating system	Exact source	Courtship types
*Nanorana venbtripunctata*	3	35.5 ± 0.5	4.3 ± 0.9	14.03 ± 1.10	0.05 ± 0.01	0.19 ± 0.03	6.92 ± 1.35	2.77 ± 0.12	0.16 ± 0.39	18.2 ± 5.3	1	3	1
*Nanorana parkeri*	3	54.7 ± 1.0	17.5 ± 1.6	17.86 ± 2.37	0.16 ± 0.01	0.74 ± 0.11	7.23 ± 1.17	3.34 ± 0.37	0.23 ± 0.02	37.3 ± 5.6	1	2	1
*Chaparana quadrana*	3	71.1 ± 3.9	43.3 ± 6.6	33.60 ± 2.17	0.42 ± 0.03	0.43 ± 0.12	12.35 ± 0.67	7.49 ± 1.33	0.48 ± 0.07	1556.7 ± 444.4	2	3	1
*Bombina maxima*	9	58.1 ± 1.1	19.0 ± 1.2	23.59 ± 1.25	0.27 ± 0.03	1.05 ± 0.09	13.16 ± 0.78	2.59 ± 0.14	0.27 ± 0.06	197.1 ± 72.9	1	3	2
*Branchytarsophry*s *chuannanensis*	2	101.9 ± 7.6	100.8 ± 12.9	43.23 ± 0.54	1.37 ± 0.86	0.94 ± 0.07	19.62 ± 0.41	6.55 ± 0.50	0.74 ± 0.07	1280.0	1	3	2
*Branchytarsophrys feae*	1	95.6	93.5	23.11	1.26	1.73	7.28	3.46	0.47	748.7	1	3	2
*Megophrys shapingensis*	2	72.6 ± 2.1	29.9 ± 1.3	22.55 ± 0.62	0.61 ± 0.22	0.74 ± 0.28	9.51 ± 1.32	3.28 ± 0.77	0.40 ± 0.02	1291.4 ± 377.0	1	3	2
*Scutiger muliensis*	2	62.5 ± 0.4	29.5 ± 3.7	33.86 ± 5.91	1.13 ± 0.05	1.83 ± 0.02	14.45 ± 2.61	3.98 ± 0.84	0.33 ± 0.09	72.9 ± 0.5	1	1	2
*Kaloula verrucosa*	5	39.2 ± 0.9	6.5 ± 0.4	20.02 ± 1.22	0.38 ± 0.02	0.77 ± 0.10	9.23 ± 0.79	3.02 ± 0.40	0.35 ± 0.03	375.3 ± 24.3	1	3	2
*Kaloula rugifera*	2	38.9 ± 1.3	7.6 ± 0.7	8.18 ± 0.17	0.08 ± 0.01	0.71 ± 0.03	4.21 ± 0.22	1.10 ± 0.17	0.30 ± 0.01	14.8 ± 1.5	1	3	2
*Rana chaochiaoensis*	6	50.4 ± 2.6	9.5 ± 1.3	22.87 ± 1.53	0.25 ± 0.004	0.74 ± 0.13	6.53 ± 0.55	5.38 ± 0.59	0.31 ± 0.05	13.8 ± 4.1	1	3	1
*Hyla tsinlingensis*	3	32.1 ± 0.5	2.3 ± 0.2	7.99 ± 0.17	0.05 ± 0.01	0.41 ± 0.03	3.29 ± 0.27	1.39 ± 0.04	0.18 ± 0.04	8.6 ± 1.8	1	3	2
*Hyla annectans chuanxiensis*	3	33.0 ± 2.6	3.2 ± 0.4	9.02 ± 1.06	0.04 ± 0.01	0.34 ± 0.03	4.82 ± 0.68	0.96 ± 0.24	0.04 ± 0.003	16.4 ± 3.4	2	3	2
*Hyla annectans jingdongensis*	6	33.5 ± 0.9	2.2 ± 0.3	11.43 ± 0.58	0.01 ± 0.06	0.45 ± 0.04	5.93 ± 0.33	2.18 ± 0.20	0.36 ± 0.02	9.4 ± 3.5	2	3	2
*Bufo melanosctictus*	1	54.6	20.35	25.96	0.44	1.22	13.73	3.26	0.50		1	3	1
*Rhacophorus omeimontis*	7	59.8 ± 1.7	11.6 ± 0.8	44.69 ± 2.88	0.31 ± 0.04	0.99 ± 0.16	15.81 ± 0.75	9.79 ± 0.79	0.90 ± 0.16	356.1 ± 68.1	2	2	2
*Polypedates megacephalus*	6	43.6 ± 1.2	5.4 ± 0.4	30.99 ± 1.91	0.20 ± 0.07	0.44 ± 0.07	10.64 ± 0.81	9.72 ± 0.73	0.87 ± 0.25	62.5 ± 16.3	2	2	2
*Rhacophorus chenfui*	2	38.6 ± 0.7	5.8 ± 0.7	17.7 ± 72.96	0.09 ± 0.02	0.60 ± 0.23	7.92 ± 1.00	3.42 ± 0.43	0.26 ± 0.01	158.5 ± 25.5	2	3	2
*Rhacophorus dugritei*	6	42.2 ± 0.6	5.4 ± 0.2	23.18 ± 0.83	0.07 ± 0.01	0.31 ± 0.03	10.64 ± 0.40	4.43 ± 0.35	0.45 ± 0.04	96.9 ± 10.6	2	3	2
*Hylarana guentheri*	20	58.8 ± 0.7	17.5 ± 0.6	40.49 ± 1.68	1.01 ± 0.10	1.06 ± 0.08	14.24 ± 0.77	10.47 ± 0.49	0.67 ± 0.04	55.9 ± 2.3	1	2	2
*Pelophylax plancyi*	2	55.3 ± 1.6	22.6 ± 1.5	27.41 ± 0.78	0.54 ± 0.08	0.43 ± 0.02	8.70 ± 0.13	7.39 ± 0.22	0.23 ± 0.03	22.1	1	3	2
*Microhyla ornata*	4	20.6 ± 0.4	0.7 ± 0.1	4.90 ± 0.32	0.01 ± 0.00	0.06 ± 0.02	2.56 ± 0.13	0.76 ± 0.03	0.06 ± 0.01	1.3 ± 0.3	1	3	2
*Oreolalax rugosus*	1	52.7	9.45	33.56	0.25	1.22	15.68	2.94	0.72	40.8	1		2
*Fejervarya limnocharis*	5	38.1 ± 1.3	5.1 ± 0.5	21.07 ± 1.23	0.12 ± 0.06	0.68 ± 0.11	7.66 ± 0.55	4.61 ± 0.23	0.26 ± 0.07	20.9 ± 2.9	1	2	2
*Amolops lifanensis*	3	52.8 ± 1.6	14.4 ± 0.9	28.84 ± 3.48	0.54 ± 0.18	0.45 ± 0.07	9.07 ± 1.15	5.29 ± 0.82	0.48 ± 0.08	44.8 ± 19.8	1	2	1
*Amolops mantzorum*	8	53.8 ± 1.2	14.2 ± 0.4	29.73 ± 2.30	0.33 ± 0.08	0.89 ± 0.18	10.47 ± 1.41	5.33 ± 0.77	0.39 ± 0.08	24.6 ± 2.6	1	2	1
*Amolops loloensis*	2	52.4 ± 0.8	18.6 ± 1.2	35.81 ± 1.96	0.27 ± 0.07	1.38 ± 0.18	14.27 ± 0.04	6.82 ± 0.26	0.64 ± 0.04	47.2 ± 3.0	1	1	1
*Amolops granulosus*	1	32.6	2.2	13.37	0.12	0.22	3.53	3.66	0.67	12.3	1	1	1
*Rana omeimontis*	9	48.0 ± 1.6	11.7 ± 1.4	39.42 ± 2.74	0.87 ± 0.11	1.28 ± 0.20	13.14 ± 1.42	10.99 ± 1.20	0.77 ± 0.09	1.6 ± 0.1	1	2	1
*Rana kukunoris*	6	45.6 ± 1.8	9.2 ± 1.0	21.11 ± 1.23	0.15 ± 0.02	1.10 ± 1.18	8.24 ± 0.63	3.51 ± 0.28	0.22 ± 0.02	20.7 ± 4.2	1	1	1
*Rana japonica*	3	52.7 ± 2.9	14.6 ± 3.8	21.04 ± 1.89	0.38 ± 0.11	0.71 ± 0.31	8.58 ± 1.71	3.45 ± 1.21	0.50 ± 0.10	29.9 ± 20.6	1	3	1
*Odorrana margaretae*	2	72.0 ± 0.6	33.2 ± 0.7	61.82 ± 1.01	0.63 ± 0.19	1.93 ± 0.46	19.96 ± 1.82	16.98 ± 1.24	0.67 ± 0.09	64.9 ± 8.2	1	2	2
*Odorrana hejiangensis*	3	46.6 ± 1.1	7.4 ± 0.5	20.74 ± 1.53	0.35 ± 0.01	0.65 ± 0.15	7.94 ± 0.85	4.47 ± 0.23	0.33 ± 0.04	16.3 ± 1.4	1	3	2
*Odorrana grahami*	7	67.6 ± 2.5	27.5 ± 3.4	55.83 ± 2.28	1.24 ± 0.14	1.66 ± 0.14	18.4 ± 01.31	11.68 ± 0.53	1.02 ± 0.12	65.0 ± 9.5	1	3	2
*Bufo gargarizans*	6	99.5 ± 3.6	88.0 ± 1.0	66.86 ± 3.55	1.29 ± 0.26	4.75 ± 0.41	30.6 ± 2.22	8.87 ± 0.58	0.92 ± 0.08	315.0 ± 22.3	1	2	1
*Bufo minshanicus*	4	67.5 ± 0.8	36.6 ± 1.6	30.36 ± 3.01	1.10 ± 0.33	1.57 ± 0.30	12.29 ± 1.62	4.85 ± 0.63	0.66 ± 0.03	161.7 ± 32.0	1	3	1
*Bufo andrewsi*	10	75.6 ± 1.3	24.4 ± 1.2	61.55 ± 2.12	0.89 ± 0.11	2.91 ± 0.22	31.41 ± 1.18	6.69 ± 0.38	1.13 ± 0.13	114.4 ± 14.3	1	2	1
*Bufo tibetanus*	2	58.6 ± 5.8	21.5 ± 8.4	25.87 ± 0.78	0.40 ± 0.02	0.84 ± 0.19	15.53 ± 0.21	2.65 ± 0.35	0.30 ± 0.04	37.0	1	3	1
*Hylarana daunchina*	3	43.7 ± 0.2	9.2 ± 0.2	19.66 ± 1.13	0.15 ± 0.04	0.48 ± 0.08	6.70 ± 0.31	4.74 ± 0.46	0.29 ± 0.05	13.0 ± 3.8	1	1	2
*Pelophylax pleuraden*	10	45.4 ± 0.8	8.4 ± 0.6	25.76 ± 3.18	0.15 ± 0.08	0.48 ± 0.09	10.12 ± 1.18	6.71 ± 1.08	0.40 ± 0.07	30.9 ± 3.2	1	2	2
*Pelophylax nigromaculata*	15	67.9 ± 2.2	28.3 ± 2.3	39.70 ± 2.50	0.19 ± 0.01	0.42 ± 0.04	17.71 ± 1.32	6.44 ± 0.57	0.92 ± 0.08	33.4 ± 3.1	1	2	2
*Paa boulengeri*	2	73.5 ± 6.3	52.7 ± 10	41.57 ± 0.45	3.44 ± 0.11	1.89 ± 0.14	12.93 ± 1.48	7.60 ± 0.42	0.89 ± 0.20	448.3 ± 1.3	1	3	2
*Paa yunnanensis*	2	60.6 ± 4.8	30.0 ± 4.1	41.07 ± 7.54	2.29 ± 1.05	1.90 ± 0.10	12.87 ± 2.45	7.05 ± 0.08	1.00 ± 0.18	660.9 ± 101.9	1	3	2

Mating system and courtship types experienced by different species. Mating system was classified on a two‐point scale: 1 = polyandry, 2 = monandry; courtship types were classified on a two‐point scale: 1 = courtship calls, 2 = searching mates. The exact source of each mating system description: 1 = field observations, 2 = reference of Liao et al. (2013), 3 = reference of Zeng et al. (2014).

### 2.2 Brain measurements

**Figure 1:**
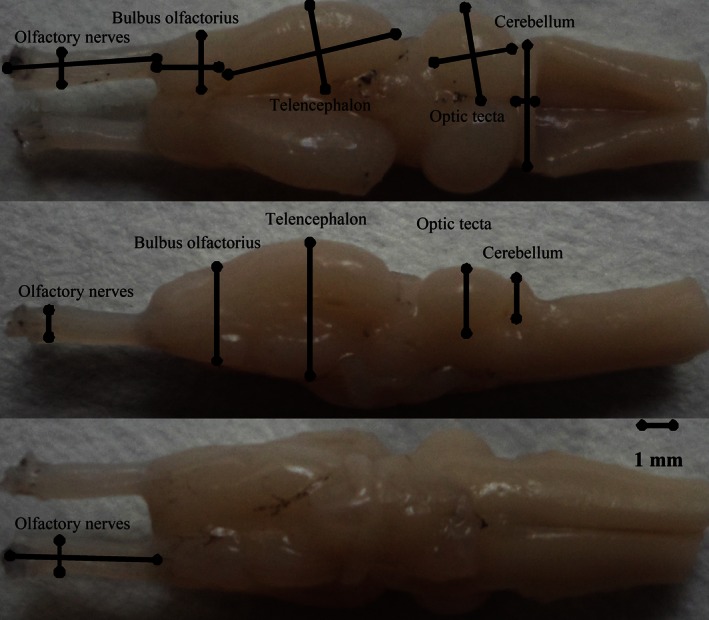
Dorsal, ventral, and lateral views of frog brain. Shown are the measures (length, width, and height) that were taken from each of the five brain structures (viz. olfactory nerves, olfactory bulbs, telencephalon, optic tectum, and cerebellum)

All dissections, digital imaging, and measurements were performed by two persons (LSL and LWB). All measurements were taken with the experimenter blind to the species identity because specimens were coded by uninformative ID‐number. We used a Motic Images 3.1 digital camera mounted on a Moticam 2006 light microscope at a 400× magnification to take digital images of the dorsal, ventral, left, and right sides of the brain and brain regions. For dorsal and ventral views, we ensured that the view of the brain being photographed was horizontal and that the brain was symmetrically positioned such that one hemisphere did not appear larger than the other. For paired regions, we only measured the width of the right hemisphere and doubled the volume estimate. We used a tpsDig 1.37 software to measure length (*L*), width (*W*), and height (*H*) of the brain and the five brain regions from the digital photographs. Brain and brain regions were defined as the greatest distance enclosed by the given region, and the used landmarks are shown in Fig. 1. Finally, we used an ellipsoid model: volume = (L*W*H) π/(6*1.43) to obtain the volumetric estimates of different brains (see details in Liao et al., 2015). For 43 species, both intrameasurer and intermeasurer repeatabilities of the intermeasurer repeatability for all brain traits are very high (Liao et al., 2015). Average brain size and average size of brain regions were used in all analyses. Before all analyses, all variables were log_10_‐transformed to meet distributional assumptions. Because some of the measurements were smaller than one, all data were multiplied by 1000 prior to log transformation (Sokal & Rohlf, 1995). We found no evidence for heterogeneity in variability across the five brain regions (Liao et al., 2015). All data are deposited on Dryad (doi:10.5061/dryad.j4754 ).

### 2.3 Data analyses

Following Zeng et al. (2014), mating system for each species was classified as: 1 = polyandry—two or more males simultaneously releasing sperm or sequentially releasing sperm in a time frame that allows for the occurrence of sperm competition; 2 = monandry—a females mates with one male over the course of a breeding season by depositing part of a single clutch. The courtship types were classified as: 1 = courtship calls—males have well‐developed vocal sacs and attract mates through their vocalization; 2 = searching mates—males do not have well‐developed vocal sacs and search for females or eggs. The classification of different species to different categories can be found in Table 1 based on the references (Liao, Zeng, & Yang, 2013; Zeng et al., 2014) and our own observation. We used dichotomous variables because for most species detailed descriptions of mating behavior are unavailable.

**Figure 2:**
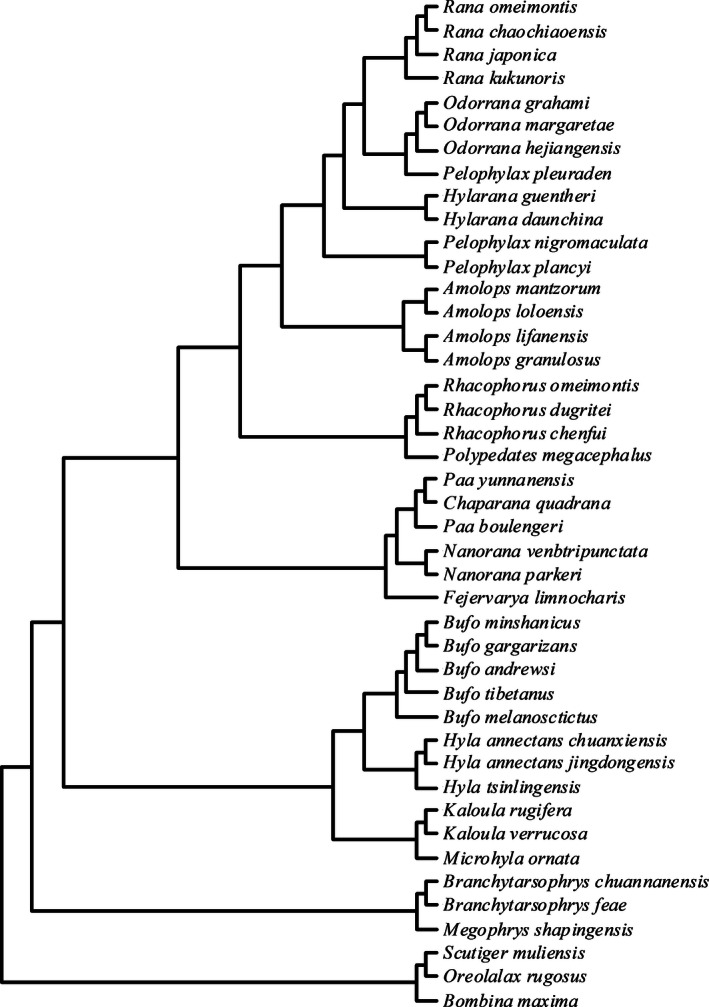
The phylogenetic tree of the 43 anurans species used in the comparative analysis following Pyron and Wiens (2011). Also see Liao et al. (2015)

For our comparative analysis, we used the phylogeny of Pyron and Wiens (2011) to reconstruct a phylogenetic tree for the 43 species (Fig. 2). The relationships between (log) brain size, size of five brain regions, and three indicators of sexual selection (i.e., mating system, type of courtship, and testes mass) were assessed in a series of phylogenetically controlled linear models. To account for the evolutionary relationships among species, we performed phylogenetically controlled generalized least‐squared (PGLS) regression analyses (Martins and Hansen 1997) using log‐transformed data in the APE‐package (R Development Core Team 2011) in R software package (V.2.13.1; Paradis, Claude, & Strimmer, 2004). The PGLS regression estimates a phylogenetic scaling parameter λ using maximum‐likelihood method. The parameter λ estimates the effect of phylogenetic signal on the relationship between brain size and other factors analyzed (λ = 0 indicating no phylogenetic signal, and λ = 1 indicating strong phylogenetic signal). We found strong phylogenetic signals for all traits examined in our study (λ: brain siz = 0.426, olfactory nerves = 0.377, olfactory bulbs = 0.358, telencephalon = 0.382, optic tectum = 0.640, and cerebellum = 0.315). As brains are subject to a wide range of selective pressures that act simultaneously, the relationships between both brain and brain regions and sexually selected traits were assessed using multiple regressions in phylogenetic ANOVAs with body size added as a covariate in all analyses to account for allometric effects.

## 3 Results

**Figure 3:**
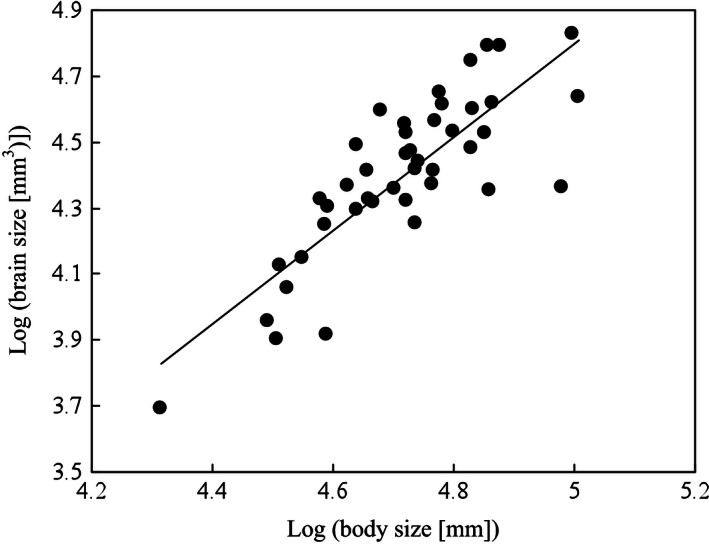
Scaling of the total brain size as functions of body size across 43 anuran species

**Figure 4:**
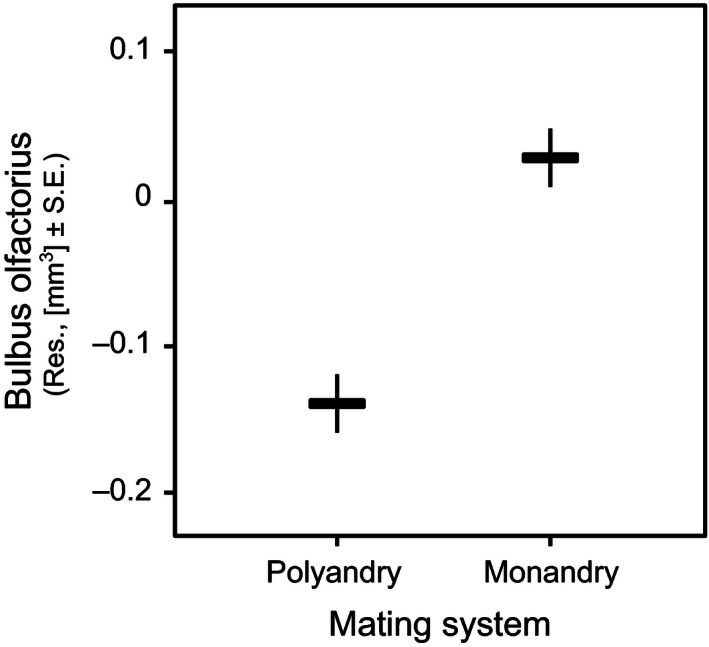
Differences in mean relative bulbus olfactorius size as a function of mating system across 43 anurans species using data corrected for phylogenetic effects. The plotted values refer to residuals from regression of bulbus olfactorius size on body size

**Figure 5:**
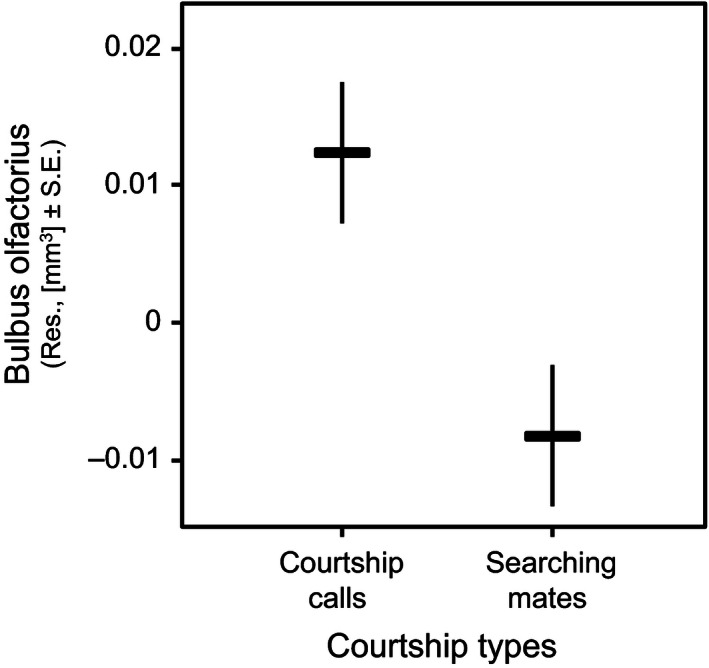
The mean relative bulbus olfactorius size in courtship types in 43 anurans species when correcting phylogenetic effects. The plotted values refer to residuals from regression of bulbus olfactorius size on body size

Across all species of amphibians tested, brain size was positively correlated with body size when correcting phylogenetic effects (slope = 3.65, *t* = 5.85, *p* < .001; Fig. 3). When controlling for body size, none of the sexually selected traits (mating system, type of courtship, testes mass) were significantly related to the amount of variation in relative brain size, and the same was true also in the case of the size of olfactory nerves, optic tecta, telencephalon, and cerebellum (Table 2). However, the size of the olfactory bulbs was significantly associated with mating system, being larger in monandrous than in polyandrous species (Table 2; Fig. 4). Olfactory bulbs size was further significantly associated with the type of courtship; calling species exhibiting larger olfactory bulbs than searching species (Table 2; Fig. 5).

**Table 2:** Regression models of (log) brain size and size of different brain structures in relation to various predictor variables for males across 43 anurans species when controlling for phylogeny (PGLS). Body size was added as a covariate and was significantly positively related to brain size and size of different brain structures in all models. The sample size, partial regression slopes (β) for the predictor variable, *t*‐ and *p*‐values are presented for each model

Source	β	*d.f*.	Predictor	*t*	*p*
Brain	−0.03250	1,43	Mating system	−0.43286	.6676
	0.03720	1,43	Courtship types	0.61762	.5406
	0.04327	1,43	Log testes mass	0.98914	.3290
	1.34431	1,43	Log body size	5.80040	<.0001
	0.01007	1,43	Number of sampling	1.27637	.2870
Olfactory nerves	−0.42542	1,43	Mating system	−1.91488	.0633
	0.26184	1,43	Courtship types	1.46829	.1505
	0.00265	1,43	Log testes mass	0.02047	.9838
	3.57151	1,43	Log body size	5.20755	<.0001
	−0.00224	1,43	Number of sampling	−0.17103	.8651
Olfactory bulbs	−0.28688	1,43	Mating system	−2.21295	.0331
	0.12004	1,43	Courtship types	2.15353	.0256
	0.00961	1,43	Log testes mass	0.12719	.8995
	2.10592	1,43	Log body size	5.26218	<.0001
	−0.00232	1,43	Number of sampling	−0.30353	.7632
Telencephalon	0.00140	1,43	Mating system	0.01767	.9860
	−0.00206	1,43	Courtship types	−0.03236	.9744
	0.05481	1,43	Log testes mass	1.18666	.2429
	1.18870	1,43	Log body size	4.85819	<.0001
	0.01362	1,43	Number of sampling	1.91736	.1624
Optic tecta	0.01854	1,43	Mating system	0.17360	.8631
	0.05349	1,43	Courtship types	0.62404	.5364
	0.08723	1,43	Log testes mass	1.40183	.1693
	1.08735	1,43	Log body size	3.29851	.0022
	0.01048	1,43	Number of sampling	1.66567	.1042
Cerebellum	−0.11904	1,43	Mating system	−0.76493	.4492
	0.09630	1,43	Courtship types	0.77094	.4456
	−0.04524	1,43	Log testes mass	−0.4989	.6208
	1.53099	1,43	Log body size	3.18686	.0029
	0.01563	1,43	Number of sampling	1.70504	.0966

## 4 Discussion

Here, we find no evidence that three prominent aspects of sexual selection are related to the overall brain size of 43 species of amphibians. However, both mating system and type of courtship influenced brain anatomy on a finer scale, albeit partly opposite to our predictions. The olfactory bulbs were larger in monandrous species and species that use calls during courtship.

The social brain hypothesis (Dunbar, 1998; Dunbar & Shultz, 2007) could be applied to predict an association between brain size and anuran mating system. It states that higher social complexity selects for larger brains because they should enable individuals to better cope with the cognitive challenges of intricate social situations. Hence, polyandrous anuran species with much shorter interaction time between individuals could be expected to show relatively smaller brains than monogamous species that usually spend extended periods of time together. This may be seen as an analogy to what has been reported in birds, where species with long‐term bonds or more complex social structures face higher cognitive demands and therefore show larger brain size (Shultz & Dunbar, 2010). In this study, however, we did not find a significant association between brain size and mating system. If this negative result holds true, we may speculate that differences in brood care could underlie this discrepancy between birds and amphibians. While monogamous birds generally also show extended periods of brood care, likely allowing the offspring to develop a larger brain, the anurans in our study do not show brood care. Whether this is the case should be determined by investigating brain morphology in brood‐caring anurans.

In contrast to the whole brain, the size of the olfactory bulbs was influenced by mating system. A larger olfactory center is commonly associated with higher olfactory acuity (Kotrschal, van Staaden, & Huber, 1998). The fact that we found larger bulbs in monandrous, compared to polyandrous species, was unexpected but may be explained by a prominent role of olfaction in anuran mate choice (Chivers, Kiesecker, & Blaustein, 1998). The advantage of choosing better mates due to better olfactory acuity during male mate choice could drive the evolution of olfactory bulb size (Verrell, 1985). Alternatively olfactory bulb evolution in monandrous animals may be driven via selection on female olfactory ability during mate choice (Candolin, 2003); the larger olfactory bulbs we observe may be the consequence of the males' and females' brains inability to evolve independently from each other within species (Finlay et al., 2001; but see Kotrschal, Räsänen, Kristjánsson, Senn, & Kolm, 2012). Future studies in female brain size and anatomy are needed to determine whether the larger olfactory bulbs are also found in monandrous females.

The second effect of sexual selection on the olfactory bulbs was opposite to our predictions; we found smaller bulbs in species searching for mates than in species using courtship calls. Whether this is directly related to searching/producing courtship calls or driven by some unknown third factor is currently unclear and will be investigated in upcoming studies.

Signals produced during courtship behavior often provide cues on male reproductive status and quality (Duellman & Trueb, 1986). More complex signals should be cognitively demanding to produce, and sexual selection may so lead to the coevolution of the size of the involved brain regions and for instance the level of complexity or the presence/absence of courtship calls. Indeed, in bird species with more complex song structure, the areas related to song production are larger (Devoogd, Krebs, Healy, & Purvis, 1993). Even the evolution of the unusually large human brain may have been driven by complex signals of courtship such as art, humor, or music (Miller, 2000). Although anuran courtship calls are not directly comparable to those complex, often learned, vocalizations of bird and mammals, they are produced by motor pattern generators in the brain (stem) (Satou, Matsushima, Kusunoki, Oka, & Ueda, 1981), we had therefore expected that whether or not a species relied on courtship calls during mate acquisition would be reflected in its brain size. While brain stem data were not available, we did not find such a difference in whole brain mass. Upcoming experiments will therefore specifically target brain stem volumes.

It is evident that analogous to ecological factors (Liao et al., 2015), the level of promiscuity can impose selection on specific brain regions in anurans. Interestingly, in contrast to those ecological factors, which impact several brain regions, sexual selection seems to only affect the olfactory bulbs. Both those results support the mosaic hypothesis of brain evolution and are therefore in line with a range of studies in other taxa finding evidence for this hypothesis (e.g., fish (Gonzalez‐Voyer et al., 2009), birds (Iwaniuk, Dean, & Nelson, 2004), or mammals (Barton & Harvey, 2000)).

Finally, the expensive sexual tissue hypothesis predicts that intense sexual selection should constrain the evolution of larger brains due to energetic trade‐offs with sexual traits. In species with high levels of sperm competition, as in many amphibians, the size of the testes provides an adequate indicator of the level of the intensity of sexual selection (Hosken & Ward, 2001). The fact that we did not find a negative association between testes mass and brain size in our study, however, does not support this hypothesis. While this lack of association may not be surprising due to the relatively small testicular volume of anurans (Liao et al., 2015), taken together with the lack of association of brain size with mating system and type of courtship, it becomes evident that for the aspects we investigated, the expensive sexual tissue hypothesis is implausible for brain size evolution in the anurans here investigated. Potentially more fine‐scaled proxies of sexual selection, such as sex ratio during courtship/egg laying or mating effort, may reveal such relationships in future studies.

In conclusion, while traits of sexual selection appear to be unrelated to brain size evolution, aspects of brain anatomy such as the olfactory bulbs are clearly shaped by both mating system and the nature of mate acquisition in male anurans.

## Funding Information

National Natural Sciences Foundation of China, (Grant/Award Number: 31471996) Austrian Science Fund, (Grant/Award Number: J 3304‐B24) Sichuan Province Outstanding Youth Academic Technology Leaders Program, (Grant/Award Number: 2013JQ0016) Sichuan Province Department of Education Innovation Team Project, (Grant/Award Number: 14TD0015, 15TD0019).

## Conflict of Interest

None declared.
